# Introduction of robotic pancreatoduodenectomy following phase 2a IDEAL guidelines

**DOI:** 10.1371/journal.pone.0302848

**Published:** 2024-05-06

**Authors:** Yoshihiro Mise, Mamiko Miyashita, Ryuji Yoshioka, Fumihiro Kawano, Yoshinori Takeda, Hirofumi Ichida, Akio Saiura

**Affiliations:** Department of Hepatobiliary-Pancreatic Surgery, Juntendo University Graduate School of Medicine, Hongo, Tokyo, Japan; Cho Ray Hospital, VIET NAM

## Abstract

**Background:**

Robotic pancreatoduodenectomy (RPD) is a newly introduced procedure, which is still evolving and lacks standardization. An objective assessment is essential to investigate the feasibility of RPD. The current study aimed to assess our initial ten cases of RPD based on IDEAL (Idea, Development, Exploration, Assessment, and Long-term study) guidelines.

**Methods:**

This was a prospective phase 2a study following the IDEAL framework. Ten consecutive cases of RPD performed by two surgeons with expertise in open procedures at a single center were assigned to the study. With objective evaluation, each case was classified into four grades according to the achievements of the procedures. Errors observed in the previous case were used to inform the procedure in the next case. The surgical outcomes of the ten cases were reviewed.

**Results:**

The median total operation time was 634 min (interquartile range [IQR], 594–668) with a median resection time of 363 min (IQR, 323–428) and reconstruction time of 123 min (IQR, 107–131). The achievement of the whole procedure was graded as A, “successful”, in two patients. In two patients, reconstruction was performed with a mini-laparotomy due to extensive pneumoperitoneum, probably caused by insertion of a liver retractor from the xyphoid. Major postoperative complications occurred in two patients. One patient, in whom the jejunal limb was elevated through the Treitz ligament, had a bowel obstruction and needed to undergo re-laparotomy.

**Conclusions:**

RPD is feasible when performed by surgeons experienced in open procedures. Specific considerations are needed to safely introduce RPD.

## Introduction

Since its description by Whipple in 1935 [1], pancreatoduodenectomy (PD) has been a technically demanding and challenging procedure that carries a high surgical risk. Notwithstanding, several pioneers have taken up the challenge to develop and promote minimally-invasive PD that may offer better quality of life compared with that in patients undergoing conventional open PD.

However, the result of a recent randomized controlled trial investigating the safety of laparoscopic PD (LPD) undermined the possibility of the easy introduction of LPD [2]. The trial was terminated because of a high 10% mortality of the patients undergoing LPD, even though the procedures were performed by trained surgeons. Recently, a robotic approach to PD was expected to overcome the limitations of LPD, with its articulated instrument arms suited to the complicated procedures of PD [3–8]; however, the multi-step procedures of robotic PD (RPD) are difficult to evaluate. The feasibility of RPD has yet to be fully assessed, which is an obstacle to the safe promotion and implementation of RPD.

In this prospective study, we present our preliminary experience of initial RPD in ten consecutive patients. A step-by-step evaluation of the procedures and modifications based on feedback from each case were demonstrated according to the Idea, Development, Exploration, Assessment and Long-term follow-up (IDEAL) guidelines (phase 2a) that are focused on the technical refinement of surgical procedures [9, 10].

## Methods

### Study design

This was a prospective, single arm study conducted at a single center. The university ethics committee approved the study protocol, and the study was registered with the University Hospital Medical Information Network Clinical Trial Registration (ID number: UMIN000037173). Written informed consent was obtained from participants. Patient data were anonymized and stored in a secure database.

### Patient cohort

**Fig 1:**
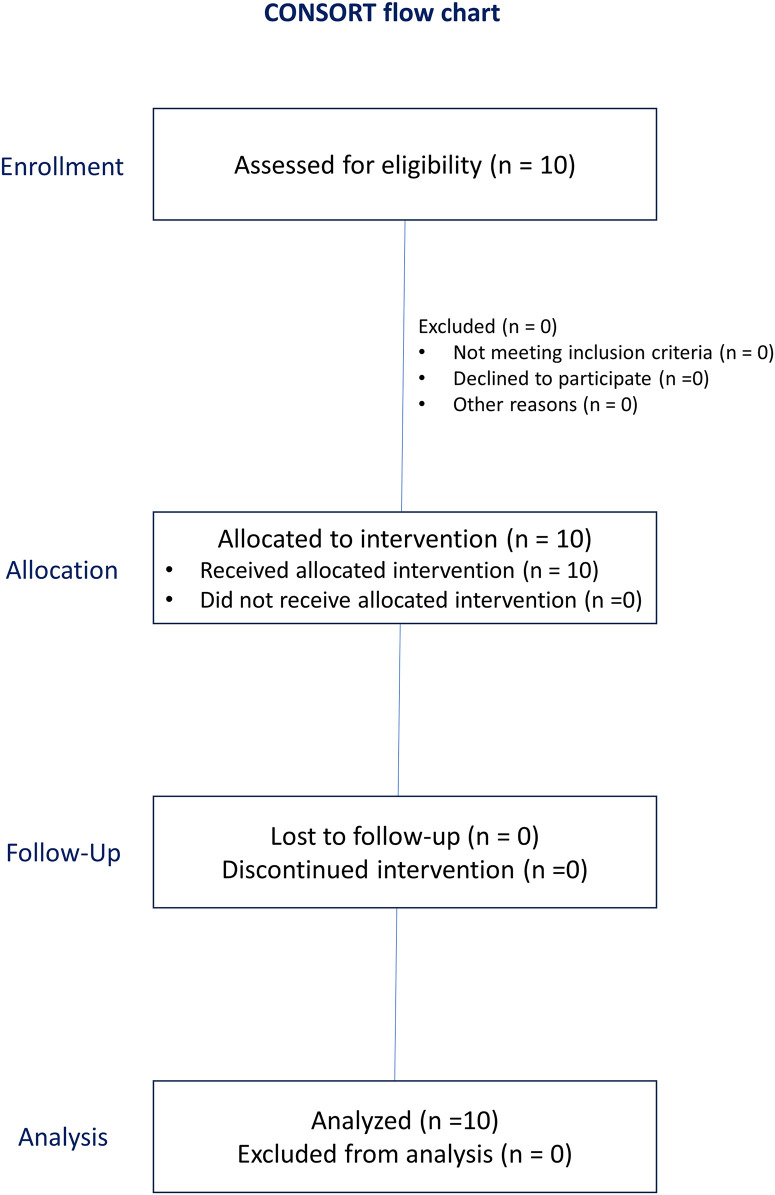
CONSORT flowchart.

The study aimed to assess ten patients who underwent RPD, in line with the IDEAL 2a framework [9]. All procedures and analyses took place at the Department of Hepatobiliary-Pancreatic Surgery of the Juntendo University Graduate School of Medicine. The recruitment period started from September 1st of 2020 and ended in November 30th of 2021 (Fig 1).

The inclusion criterion was any patient with malignant or benign tumors for which PD was scheduled. The exclusion criteria were an age less than 20 years or over 80 years, performance status of 2 or more, tumors requiring vascular reconstructions, or a contraindication for pneumoperitoneum.

### Surgical team

Two console surgeons performed the operations to minimize the variability of surgical technique (A.S. with experience of more than 500 open PDs and Y.M. with experience of more than 200 open PDs). Although the two surgeons had expertise in open PD, the number of LPDs performed was less than ten for each surgeon. Our surgical team observed several foreign and domestic institutions with greater experience in performing RPD. Notably, the surgical procedures and devices, including the type and the length of strings, were compiled in a detailed manual referring to those used at Taipei Veterans General Hospital.

Prior to the first RPD case, dry lab simulation training in pancreatico-jejunostomy (PJ) and hepatico-jejunostomy (HJ) was performed using the da Vinci Xi system (Intuitive Surgical, Sunnyvale, CA). The inanimate bio-tissue model (FASOTEC Co., Ltd., Chiba, Japan) was used for this training. The diameter of the main pancreatic duct of this model was about 1.5 mm. Additionally, three robotic distal pancreatectomies were performed before performing the first RPD case at our institution.

### Surgical procedures

**Fig 2:**
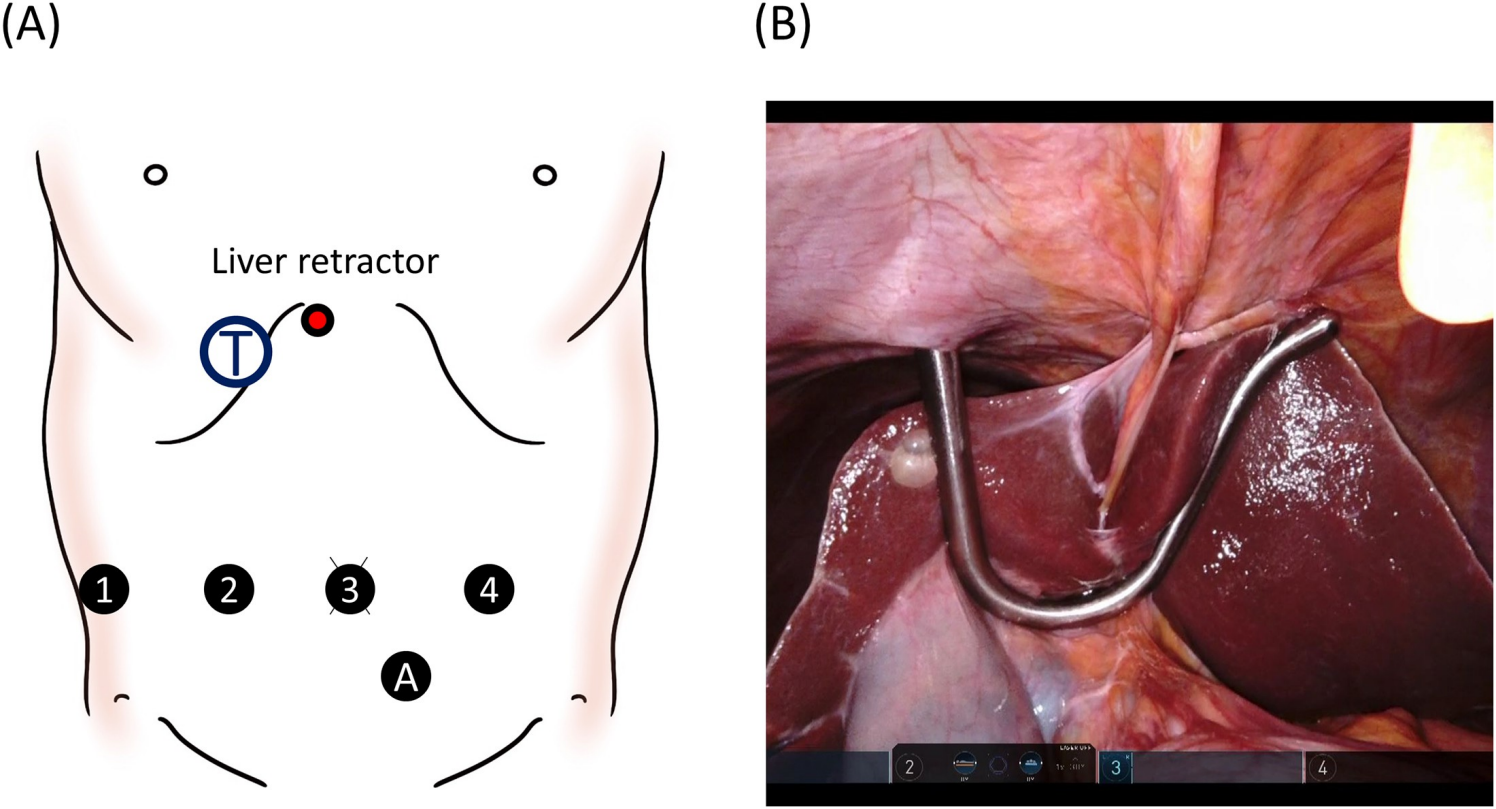
Trocar setting. (A) Robotic trocars (1–4) are placed at the level of the umbilicus. T, target; A, assistant port. (B) The liver retractor is inserted from the xyphoid prior to the docking of the robot.

Patients were placed in a reverse Trendelenburg position of 10 degrees. The trocar setting is shown in Fig 2A. Prior to docking, a liver retractor was inserted from around the xyphoid (Fig 2B).

Our RPD procedures were divided into 13 sections as follows: (1) insertion of trocars and docking of the robot; (2) division of the omentum and gastrocolic ligament; (3) mobilization of the duodenum (Kocher maneuver); (4) dissection of the left side of the superior mesenteric artery (SMA); (5) dissection around the upper edge of the head of the pancreas; (6) division of the stomach (or duodenum) and pancreas; (7) dissection around the SMA and the superior mesenteric vein (SMV); (8) dissection of the hepatoduodenal ligament; (9) harvest of the specimen; (10) PJ anastomosis; (11) HJ anastomosis; (12) retrieval of the specimen at a mini-laparotomy through a 5-cm incision of the umbilicus, then gastro (or duodeno)-jejunostomy (or plus Braun anastomosis) at the same incision; and (13) drain placement and wound closure. Sections 2 to 11 were planned to be performed using the da Vinci robotic system.

The methods of PJ and HJ anastomosis, which followed the Taipei Veterans General Hospital procedures, were standardized via dry lab training. PJ was performed using the Blumgart method and duct-to-mucosa suturing. HJ was done with running suture using monofilament thread. The details of PJ anastomosis have been visually demonstrated elsewhere [11].

### Discontinuance criteria

The discontinuance criteria were set as prolonged resection time estimated to be longer than 360 min, or excessive blood loss estimated to be more than 800 mL from docking to harvest of the specimen. These criteria were defined based on the historical outcomes of all our open PD procedures, wherein the third quartiles of duration and blood loss were 558 min and 800 mL, respectively. Judgement of discontinuance was conducted by evaluators at 240 min after completion of docking.

### Evaluation and feedback

A review meeting was held before each new case, where the procedures of the previous case were discussed based on the operative records and videos. The procedures of each case were graded according to the definitions described in Table 1.

**Table 1:** Definitions of achievements for the entire procedure.

**A**	"successful"	All procedures were completed as planned or modified according to intraoperative findings with intent.				
**B**	"feasible"	Modification of procedure sequence was needed due to unintended troubles.				
		E.g.,	Dissection of hepatoduodenal ligament was conducted due to bleeding from the superior mesenteric vein during dissection around the head of the pancreas.			
		Although all the procedures were completed as planned, a mini-laparotomy was performed to confirm the intraoperative diagnosis or when robotic reconstruction was not feasible.				
**C**	"unsuccessful"	Conversion to laparotomy due to violation of discontinuance criteria without lethal intraoperative accidents.				
**D**	"unfeasible"	Conversion to laparotomy due to lethal intraoperative accidents.				

The IDEAL protocol allows refinement of surgical technique during phase 2 studies. Thus, while most of the steps were standardized prior to the first case, several modifications were made based on the evaluations [9, 10].

All clinical data were collected prospectively. Postoperative pancreatic fistula (POPF) was defined based on the International Study Group of Pancreatic Fistula definition [12]. Postoperative complications were classified according to Clavien-Dindo grade [13]. Continuous data were expressed as the median or interquartile range (IQR), which is defined as the range between the 25th and 75th percentiles of the data.

## Results

From September 2020 to November 2021, seventy-eight patients underwent PD at our institution, and ten patients were deemed eligible for the study protocol. With informed consent obtained, the ten patients underwent RPD according to the study protocol. Table 2 summarizes the characteristics and surgical outcomes of the study cohort.

**Table 2:** Patient characteristics and short-term outcomes.

	Age	Sex	BMI	Diagnosis	Tumor size (cm)	Main pancreatic duct size (mm)	Texture of the pancreas	Operation time (min)					Blood loss (mL)	Achievement	C-D grade	POPF	Hospital stay (days)
								Total	Docking	Resection	PJ+HJ	Laparotomy					
1	75	F	16	Vater ca.	0※	2	soft	674	24	430	220†		75	B	1	−	21
2	58	M	22	Vater ca.	1	3	soft	852	26	505	170	151	265	A	2	−	16
3	44	F	28	NET	1	2	soft	601	32	293	128	148	55	A	2	B	32
4	58	F	17	NET	1	2	soft	627	39	325	140	123	10	A	2	B	34
5	73	M	22	IPMN	3	3	soft	591	27	340	110	114	140	A	3a	−	36
6	74	F	19	PDAC	in situ	4	soft	651	27	423	118	83	110	A	3b	−	42
7	70	M	22	PDAC	in situ	5	hard	592	84	322	93	93	110	A	2	−	10
8	79	F	18	Vater ca.	2.5	3	soft	578	80	319	97	82	110	A	2	−	20
9	78	F	21	Distal bile duct ca.	3	3	soft	640	75	385	180†		160	B	2	B	42
10	71	M	28	PDAC	1.5	4	hard	880	27	605	127	121	402	A	2	−	24
Median	72		22					634	30	363	123	118	110				28

^†^Patients #1 and #9 underwent reconstruction at a mini-laparotomy because of hypercapnia following pneumoperitoneum

^※^ Endoscopic resection of Vater carcinoma of patients #1 resulted in tumor exposure. No residual tumor was found in the specimen after surgery.

BMI, body mass index; C-D, Clavien-Dindo; POPF, postoperative pancreatic fistula; PJ, pancreato-jejunostomy; HJ, hepatico-jejunostomy; NET, neuroendocrine tumor; IPMN, intraductal papillary mucinous neoplasm; PDAC, pancreatic ductal adenocarcinoma

**Fig 3:**
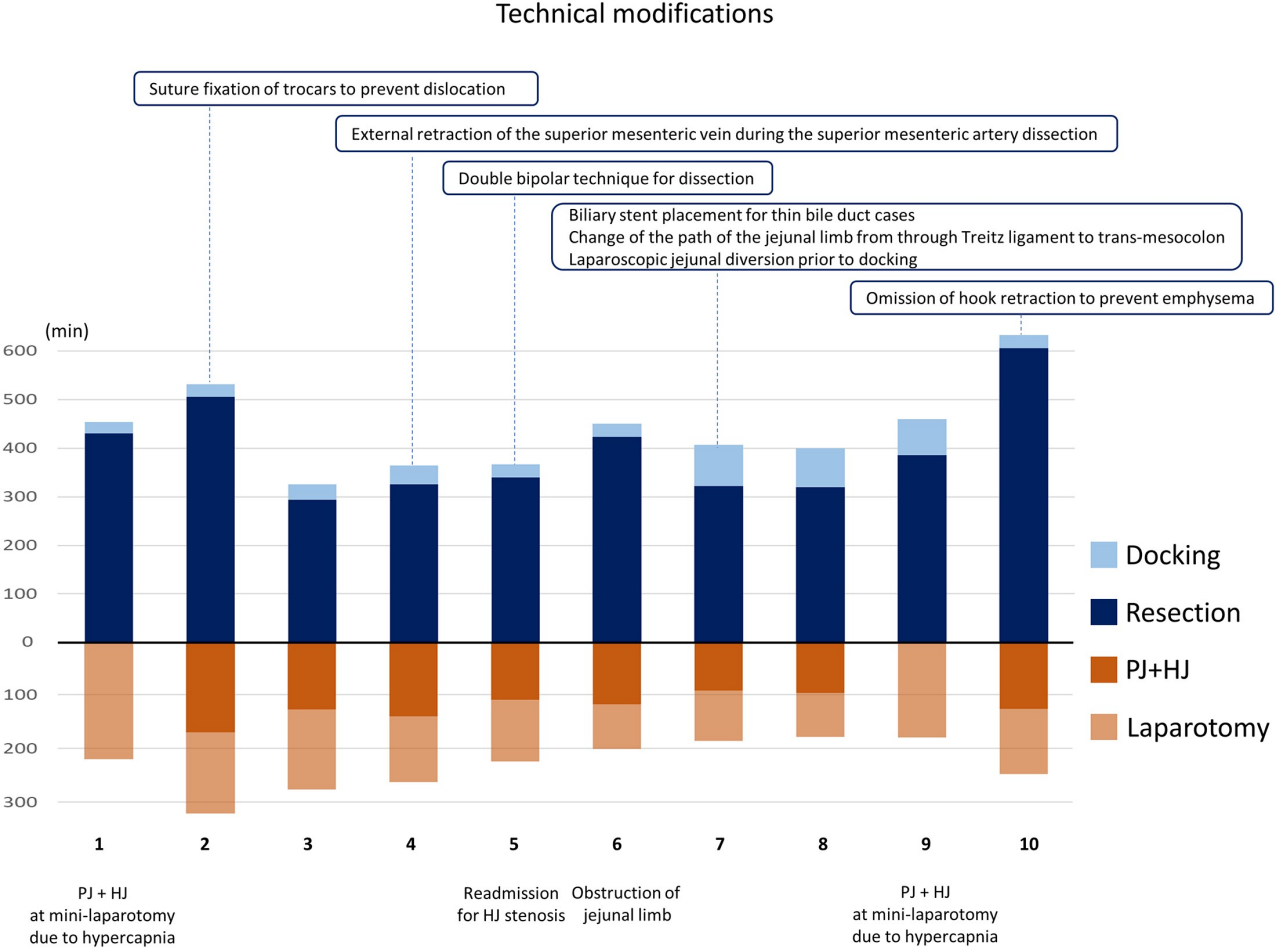
Operation time and technical modifications following refinements in each case. PJ, pancreatico-jejunostomy; HJ, hepatico-jejunostomy.

The median duration of total operation, docking (section 1), resection (sections 2 to 11), PJ+HJ (sections 10 and 11), and laparotomy (sections 12 and 13) were 634 (interquartile range [IQR], 594–668), 30 (IQR, 27–66), 363 (IQR, 323–428), 123 (IQR, 107–131), and 118 min (IQR, 91–129), respectively. The median amount of blood loss was 110 mL (range, 10–402 mL). In all patients, the resection was completed robotically without converting to laparotomy. However, patients #1 and #9 underwent PJ and HJ at a mini-laparotomy because of hypercapnia following extensive pneumoperitoneum. Operation times and technical modifications following evaluation and feedback from each case are summarized in Fig 3.

Major postoperative complications defined as Clavien-Dindo grade 3 or higher occurred in two patients. Three patients were classified as grade B POPF because the duration of surgical drains reached beyond three weeks. However, no specific treatment was needed in these patients. No patients had delayed gastric emptying nor postpancreatectomy hemorrhage. The median length of hospital stay was 28 days (range, 10–42 days). No 90-day mortality was observed.

### Evaluation and refinements

#### Patient #1

No critical problems occurred during the resection part. However, PJ and HJ anastomosis were performed at a mini-laparotomy because of hypercapnia following extensive pneumoperitoneum detected at the end of the resection.

Major refinement: Assistant trocars dislocated several times, which may have been the cause of the pneumoperitoneum. Assistant trocars need to be fixed with suture before docking.

Minor refinement: The length of a vessel loop to encircle the bile duct should be longer (from 8 cm to 12 cm).

#### Patient #2

The resection time was over 500 min because the dissection around the hepatoduodenal ligament was technically demanding due to inflammation following repetitive cholangitis. Loss of the suture needle and the attempt to pull it out using ordinary forceps was another cause of the prolonged operation time.

Minor refinement: Suture needles should be pulled out using needle-drivers. During PJ anastomosis, the length of strings for the posterior wall suture should be longer to fix the pancreatic duct stent (from 6 cm to 8 cm).

#### Patient #3

Assistant’s forceps from the 5 mm port interfered with robotic arm #1 and the camera.

Minor refinement: The assistant 5 mm port should be placed more caudally.

#### Patient #4

External retraction of the SMV during dissection around the SMA was introduced in light of a technical report [14].

#### Patient #5

Double bipolar technique for tissue dissection was introduced after referring to technical reports [15, 16]. No critical problems occurred during the operation, and the patient was discharged on the 36th postoperative day. However, he was re-admitted because of stenosis of the HJ anastomosis on the 74th postoperative day.

Major refinement: A biliary stent should be inserted for patients that have a thin bile duct.

#### Patient #6

**Fig 4:**
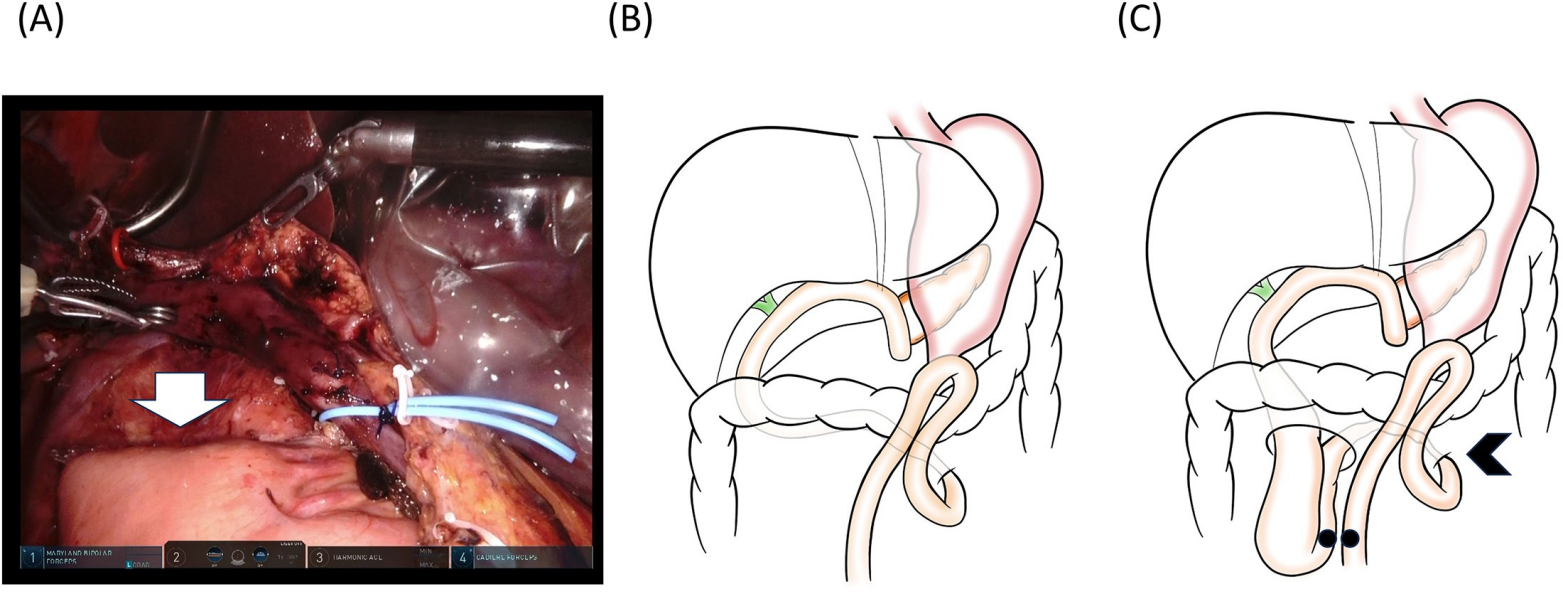
Elevation of the jejunal limb via the “Treitz” route led to bowel obstruction in patient #6. (A) After resection, the cut end of the jejunum was easily found (arrow). (B) Reconstruction using the jejunal limb elevated via “Treitz” route. (C) Bowel obstruction was found at the Treitz ligament (arrowhead). At re-laparotomy, bypass anastomosis was made between the sites indicated by the two black dots.

The patient suffered from obstruction of the jejunal limb placed through the ligament of Treitz. (Fig 4).

The obstructed jejunal limb was endoscopically drained. However, it failed to solve the kinking at the Treitz ligament. On the 28th postoperative day, re-operation was performed, which revealed that the jejunal limb was obstructed at the ligament of Treitz because the limb sank through a hole in the mesocolon.

Major refinement: The path of the jejunal limb was changed from through the Treitz ligament to trans-mesocolon.

#### Patient #7

Prior to docking, laparoscopic observation revealed adhesion of the jejunum around the Treitz ligament. After dissection of the adhesion, the jejunum was diverted and cut laparoscopically, which shortened the length of resection time.

Major refinement: Jejunal diversion may be better performed laparoscopically before docking.

#### Patient #9

The external SMV retraction technique was modified to retract the SMV towards the right side to directly reach the SMA from the left side of the SMV. Indocyanine green fluorescence imaging (Firefly) was effective for detecting the branch of the inferior pancreaticoduodenal artery. PJ and HJ anastomosis were performed at a mini-laparotomy because of hypercapnia following extensive emphysema detected at the end of the resection. Insertion of the hook to retract the liver was thought to be the cause of emphysema.

Major refinement: The hook to retract the liver should be inserted after resection.

#### Patient #10

Due to a high body mass index of 28 and a history of laparoscopic low anterior resection for rectal cancer, the resection took a longer time without major problems. Additionally, abnormal branching of the common hepatic artery that ran behind the SMV made the operation technically demanding at the end of the resection. Despite the prolonged resection time, no emphysema was observed due to omission of hook insertion at the xyphoid process.

## Discussion

In this study, based on the IDEAL phase 2a framework, we demonstrated that RPD can be safely introduced by a step-by-step refinement of procedures. The refinement processes of RPD revealed in this study should help surgeons to safely introduce RPD while avoiding some trials and errors. Although the operation times were longer in our case series, RPD was completed with grade A, “successful”, achievement in 8 of 10 patients. In the remaining two patients, reconstruction was converted to a mini-laparotomy due to extensive pneumoperitoneum (grade B). As for postoperative outcomes, we observed Clavien-Dindo grade 3 complications in two patients, and POPF grade B in three patients, which were comparable to the open benchmark recommendations (≤ 30% and ≤ 19%, respectively) [17].

One of the lessons learned in this study was that the path of the jejunal limb for reconstruction during RPD should not be through the ligament of Treitz. Patient #6 suffered from obstruction of the jejunum limb that kinked at the ligament of Treitz, which may have been avoided with the limb placed via the mesocolon. There are two main routes of the jejunal limb for PD reconstruction; through the right of the middle colic vessels in the transverse mesocolon, or behind the mesenteric vessels through the ligament of Treitz [18]. Evidence regarding the optimal pathway of the limb is scarce. The path through the ligament of Treitz is reportedly associated with a higher incidence of biliopancreatic limb obstruction due to local recurrence after PD for pancreatic ductal adenocarcinoma [18]. During RPD, in which dynamic mobilization of the bowel is technically demanding due to the limited aeroperitoneum space, the jejunal stump can be brought up more easily through the ligament of Treitz as shown in Fig 4A. However, the current study warns that the path through the ligament of Treitz can cause bowel obstruction unrelated to tumor recurrence at the ligament of Treitz.

Pneumoperitoneum intolerance was observed in 2 of 10 patients which resulted in conversion to a mini-laparotomy for PJ and HJ reconstruction. A recent review of 4587 RPD cases demonstrated that the conversion rate due to pneumoperitoneum intolerance was 8.3% [19]. The higher incidence in our series might have been attributable to the prolonged median operation time of 634 min. Shortening the operation time by gaining experience is apparently a key to reducing the pneumoperitoneum intolerance during RPD. Additionally, insertion of the hook to retract the liver was thought to be the cause of extensive pneumoperitoneum in our series. Although the hook retractor of the liver provides a better surgical view, especially during PJ and HJ procedures, the hook should be inserted only when needed or prior to reconstruction to avoid pneumoperitoneum intolerance.

Shortening the operation time of RPD is a challenge that needs to be addressed. A previous study of 450 RPD cases demonstrated that the RPD learning curve reaches a plateau after around 100 cases [20]. However, it was inconclusive about which of the multiple steps in RPD were difficult and time-consuming. The current study revealed that reconstruction (PJ+HJ anastomosis) time is stable at around 2 hours with small variance, as shown in Fig 3, implying that reconstruction in RPD can be standardized from the beginning if you have expertise in open procedures and have trained well at the dry lab. Hence, the key to shortening the operation time is to stabilize the resection part of RPD. Several pointers in shortening the resection time were found in this prospective study. In patient #7, adhesion around the Treitz ligament compelled us to mobilize the jejunum laparoscopically prior to the docking of the robot, which led to the shortening of the jejunal limb preparation time. Because the robot limits vertical and dynamic movements, a temporary laparoscopic approach may be effective when preparing the jejunal limb below the transverse colon. Another way to facilitate the resection part of RPD is external traction of the portal vein, as advocated by Ikoma et al [14]. This traction allows the freeing-up of one hand while dissecting around the SMA, which is the most challenging step of RPD. Further collective knowledge will contribute to the safe promotion and implementation of RPD in clinical practice.

The main limitation of this study was that these results were obtained by two surgeons that shared significant experience in open PD. However, they did not have so much experience with LPD, which indicates that RPD can be safely introduced when surgeons have expertise in open procedures and have been well-trained at the dry lab. Another limitation was that the discontinuance criterion of a resection time of 360 min was not appropriately set in this protocol. Although the evaluators judged resection would be safely completed 240 min after the resection began, the median resection time was 363 min, and 5 cases exceeded this criterion. Considering that the reported operation time of RPD ranges widely, from 188 to 718 min [19], the criterion of operation time should not have been set so rigidly.

In conclusion, our IDEAL phase 2a study demonstrates that RPD can be safely introduced in the hands of a surgical team with experience of open PD. The trials and errors demonstrated in this report will help other institutions trying to introduce RPD and to circumvent these errors.

## Supporting information

S1 Checklist TREND statement checklist. (PDF)
